# Why does the acquired cholesteatoma trigger resorption of the temporal bone?

**DOI:** 10.1007/s00405-017-4633-5

**Published:** 2017-06-14

**Authors:** Jerzy Kuczkowski, Tomasz K. Nowicki, Anna Starzyńska

**Affiliations:** 1 Department of Otolaryngology, Medical University of Gdansk, Smoluchowskiego 17, 80-214 Gdansk, Poland; 2 2nd Department of Radiology, Medical University of Gdansk, Smoluchowskiego 17, 80-214 Gdansk, Poland; 3 Department of Oral Surgery, Medical University of Gdansk, Smoluchowskiego 17, 80-214 Gdansk, Poland

To the Editor,

We have read with great interest the article “The role of bone resorption in the etiopathogenesis of acquired middle ear cholesteatoma” by Xie et al. [1]. The authors presented review article about bone resorption in acquired cholesteatoma. We would like to make some comments concerning this problem based on our experience and literature review. Osteolysis in the cholesteatoma of the ear is caused by several factors: epithelial hyperproliferation, bacterial infections, increased pressure in tympanic cavity, osteoclasts activation, RANKL/OPG imbalance, increased acidity of the environment, increased activity of bone-destroying enzymes, increased production of inflammatory mediators and other factors [2, 3]. The inflammatory cytokine is proved to be closely associated with bone resorption in chronic otitis media with cholesteatoma (ChCOM). Osteolysis is a hallmark of exacerbation ChCOM and development of intracranial/extracranial complications. Destruction of scutum, bone canal of the external auditory canal, the tympanic cavity walls, and ossicles is observed in the microscopic examination of patients with ChCOM. In the high-resolution computed tomography of the temporal bone destruction of the tympanic and mastoid cavity, bony labyrinth, facial nerve canal, ossicles, scutum, external auditory canal, and tegmental roof of the tympanic cavity can be seen (Fig. 1). Cholesteatoma proliferation with infection and accumulation of purulent content leads to increased pressure in the middle ear. Infections of the ear, especially anaerobic, favour activation of macrophages, lymphocytes, monocytes, endotheliocytes, and fibroblasts, present in the perimatrix of cholesteatoma. Activation of cells present in cholesteatomas perimatrix leads to increased excretion of inflammatory mediators. It has been proved that increased activation of osteoclasts and excretion of TNF-alpha, IL-1, and IL-6 is present in the “blocked ear” with reported radiologically osteolysis. TNF-alfa leads to bone resorption directly by stimulating the differentiation and maturation of osteoclast and indirectly by exposing the bone matrix to the action of the osteoclasts. In cholesteatoma in comparison with normal skin, we observed not only a 3- and 3.5-fold higher level of IL-1 and IL-6 accordingly, but also a strongly positive correlation between the expression of these two cytokines and the degree of bone resorption [4].

**Fig. 1:**
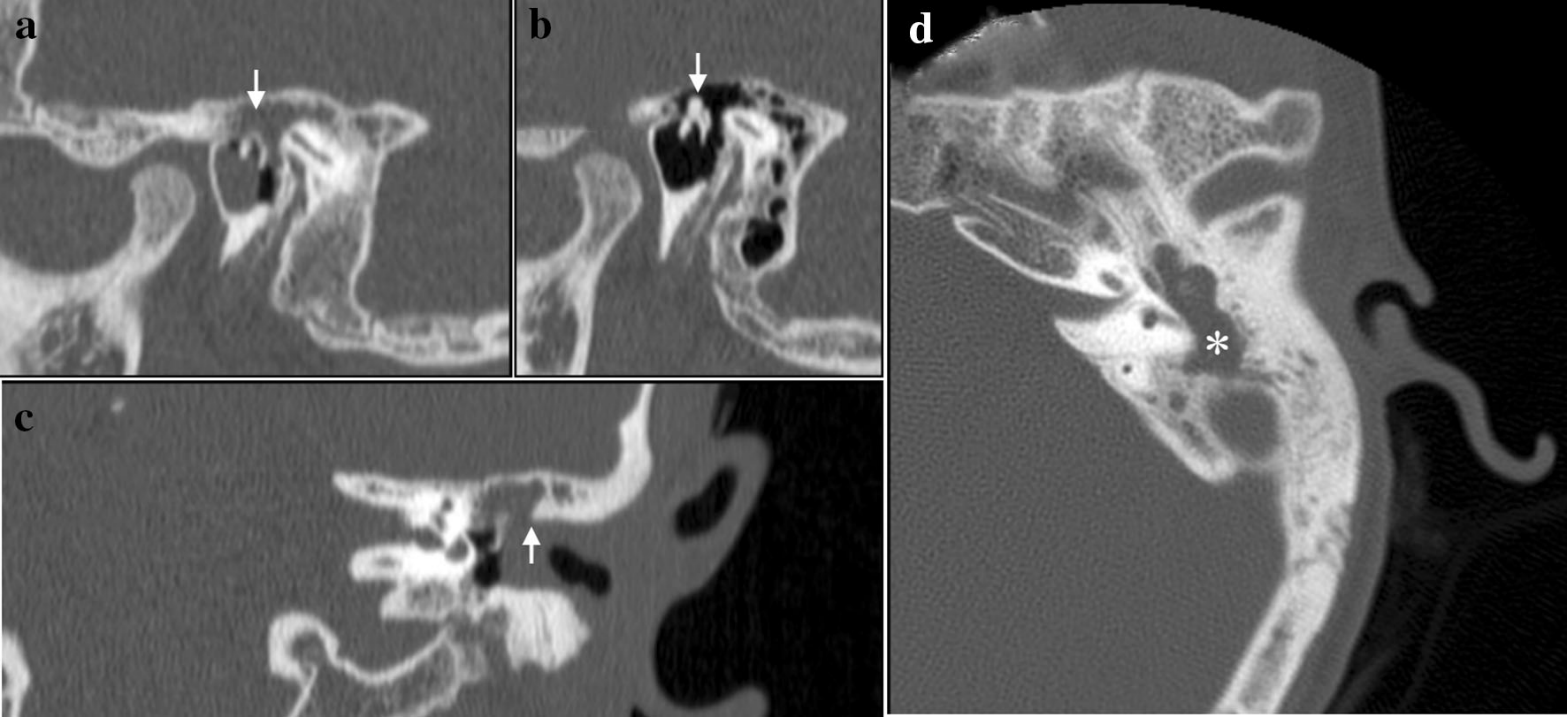
Cholesteatoma of the left middle ear in a 14-year-old boy in high-resolution CT. **a** Osteolysis of the head of the malleus and the body of the incus (*arrow*) in the sagittal plane (multiplanar reconstruction). Epitympanum is filled by soft-tissue mass (cholesteatoma). **b** Normal ear in the sagittal plane for comparison. **c** Destruction of scutum, again soft-tissue mass is visible in epitympanum (coronal plane). The ossicles are typically displaced medially. **d** Enlargement of the tympanic antrum with a soft-tissue mass within (*asterisk*)

## References

[1] Xie S, Wang X, Ren J, Liu W (2017). The role of bone resorption in the etiopathogenesis of acquired middle ear cholesteatoma. Eur Arch Otorhinolaryngol.

[2] Kuczkowski J, Kobierska-Gulida G, Iżycka-Swieszewska E, Potocka M, Mikaszewski B, Sierszeń W (2010). Molecular control of bone resorption in chronic otitis media with cholesteatoma. Otolaryngol Pol.

[3] Kuczkowski J, Sakowicz-Burkiewicz M, Iżycka-Świeszewska E (2010). Expression of the receptor activator for nuclear factor kappaB ligand and osteoprotegerin in chronic otitis media. Am J Otolaryngol.

[4] Kuczkowski J, Sakowicz-Burkiewicz M, Iżycka-Świeszewska E, Mikaszewski B, Pawełczyk T (2011). Expression of tumor necrosis factor-a, interleukin-1a, interleukin-6 and interleukin-10 in chronic otitis media with bone osteolysis. ORL J Otorhinolaryngol Relat Spec.

